# *CCND1* Amplification in Breast Cancer -associations With Proliferation, Histopathological Grade, Molecular Subtype and Prognosis

**DOI:** 10.1007/s10911-022-09516-8

**Published:** 2022-04-22

**Authors:** Marit Valla, Elise Klæstad, Borgny Ytterhus, Anna M. Bofin

**Affiliations:** 1 Department of Clinical and Molecular Medicine, Faculty of Medicine and Health Sciences, Norwegian University of Science and Technology, Trondheim, Norway; 2 Clinic of Laboratory Medicine, St. Olav’s Hospital, Trondheim University Hospital, 7006 Trondheim, Norway

**Keywords:** Breast cancer, *CCND1*, Gene amplification, Copy number, Prognosis

## Abstract

*CCND1* is located on 11q13. Increased *CCND1* copy number (CN) in breast cancer (BC) is associated with high histopathological grade, high proliferation, and Luminal B subtype. In this study of *CCND1* in primary BCs and corresponding axillary lymph node metastases (LNM),we examine associations between *CCND1* CN in primary BCs and proliferation status, molecular subtype, and prognosis. Furthermore, we studied associations between *CCND1* CN and CNs of *FGFR1* and *ZNF703*, both of which are located on 8p12. Fluorescence in situ hybridization probes for *CCND1* and chromosome 11 centromere were used on tissue microarrays comprising 526 BCs and 123 LNM. We assessed associations between *CCND1* CN and tumour characteristics using Pearson’s χ^2^ test, and estimated cumulative risks of death from BC and hazard ratios in analysis of prognosis. We found *CCND1* CN ≥ 4 < 6 in 45 (8.6%) tumours, and ≥ 6 in 42 (8.0%). *CCND1* CN (≥ 6) was seen in all molecular subtypes, most frequently in Luminal B (HER2^−^) (20/126; 16%). Increased *CCND1* CN was associated with high histopathological grade, high Ki-67, and high mitotic count, but not prognosis. *CCND1* CN ≥ 6 was accompanied by CN increase of *FGFR1* in 6/40 cases (15.0%) and *ZNF703* in 5/38 cases (13.2%). Three cases showed CN increase of all three genes. High *CCND1* CN was most frequent in Luminal B (HER2^−^) tumours. Good correlation between *CCND1* CNs in BCs and LNM was observed. Despite associations between high *CCND1* CN and aggressive tumour characteristics, the prognostic impact of *CCND1* CN remains unresolved.

## Introduction

Breast cancer (BC) is the most prevalent cancer type and the leading cause of cancer-related death among women worldwide [1]. It is also highly heterogeneous and the need for individually-tailored treatment strategies has become increasingly apparent as the short- and long-term effects of current treatment regimens emerge [2]. There is therefore a need for new prognostic biomarkers that can contribute to further fine-tune our approach to BC diagnostics and treatment.

*CCND1* is located on the long arm of chromosome 11 at 11q13.3 and encodes cyclin D1 protein [3]. Cyclin D1 is involved in cell cycle progression by inducing G1-S transition through activation of cyclin-dependent kinases, Cdk4 and Cdk6 [4, 5]. Cyclin D1 may also impact steroid hormone receptors, activating the oestrogen receptor (ER) [6, 7], and inhibiting the androgen receptor in breast epithelium [8]. While Cyclin D1 overexpression is reported in approximately 50% of BCs [9, 10], the frequency of *CCND1* amplification is between 9–15% [9, 11–13]. *CCND1* amplification is associated with increased risk of recurrence [9, 12, 13] and reduced chemosensitivity in BC [11]. There is also an association between *CCND1* amplification and high proliferation, high histopathological grade [9, 12], and the Luminal B subtype [9]. *CCND1* amplified BCs may also show concurrent amplification of *Fibroblast growth factor receptor 1* (*FGFR1)* [14], and/or *Zinc finger protein 703* (*ZNF703*) [15], both of which are located at 8p11.23.

Using fluorescence in situ hybridization (FISH), we studied *CCND1* copy number (CN) in tissue microarrays (TMA) from formalin-fixed, paraffin-embedded BC tissue (FFPE) from primary BCs and their corresponding axillary lymph node metastases. The main aim was to investigate associations between *CCND1* CN alterations in primary BC tumours and proliferation status, molecular subtype, and prognosis. A secondary aim was to study *CCND1* CN in corresponding axillary lymph node metastases. Furthermore, previous studies have shown that there may be an association between amplifications in specific regions of chromosome 8 and chromosome 11 [15, 16]. Therefore, we also aimed to see if there was an association between CNs of *CCND1*, *FGFR1* and *ZNF703.* The latter two genes are located on chromosome 8 and have previously been studied by our group in the same series of patients [17, 18]. In these studies, we found that *FGFR1* and *ZNF703* CN increase was associated with high histopathological grade, proliferation, and the Luminal B subtype. *ZNF703* CN increase was also associated with a poor prognosis.

## Materials and Methods

### Study Population

**Fig. 1:**
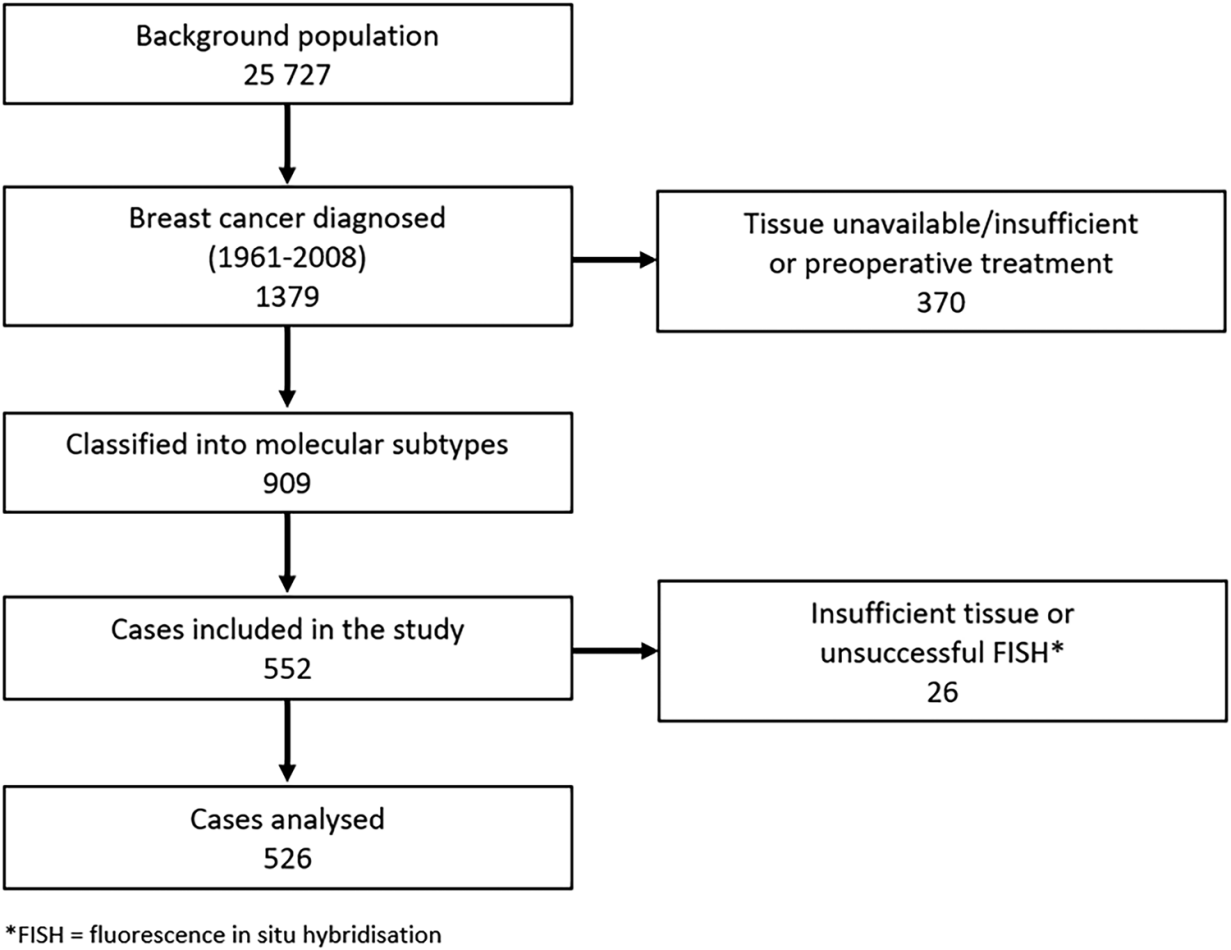
Overview of the background population and cases included in the study

In 1956–1959, a population-based study for the early clinical detection of BC was carried out in the county of Nord-Trøndelag, Norway [19]. In total, 25,727 women born between 1886–1928 were invited, and of these, 1379 were diagnosed BC during follow-up from 1961–2008. Patients were identified through linkage with the Cancer Registry of Norway, and information on date and cause of death was obtained from the Norwegian Cause of Death Registry. Of the 1379 incident cases, 909 were previously successfully reclassified into molecular subtypes [20]. In the present study, FISH was carried out on TMAs mainly containing tumour tissue diagnosed in the 1980s or later (n = 552). Of these, 13 were excluded due to insufficient amounts of tumour tissue, and 13 were excluded due to unsuccessful FISH. Thus, 526 tumours were included for assessment of *CCND1* and chromosome 11 enumeration probe (CEP11) CN in the primary tumours (Fig. 1). Of these, 177 had lymph node metastases. Tissue from lymph node metastases was available in TMAs for 132 cases, and of these, four were excluded due to insufficient tumour tissue, and five were excluded due to unsuccessful FISH. Thus, *CCND1* and CEP11 CN in lymph node metastases was registered for 123 cases.

### Specimen Characteristics

The primary tumours were previously reclassified into histopathological type and grade [20, 21]. From each case, three 1 mm in diameter tissue cores from the periphery of the primary tumour, and three 1 mm cores from the lymph node metastases were assembled in tissue microarrays. Primary tumours were then reclassified into molecular subtypes using immunohistochemistry (IHC) and chromogenic in situ hybridization (CISH) (Table 1). IHC was used for assessment of ER, PR, Ki67, CK5 and EGFR, and Both CISH and IHC were used for assessment of HER2 [20]. In the previous *ZNF703* and *FGFR1* studies, FISH was used to target the two genes and the chromosome 8 centromere, as previously described [17, 18].

**Table 1:** Molecular subtyping algorithm

Molecular subtype	Subtyping algorithm
Luminal A	ER^+^ and/or PR^+^, HER2^−^, Ki67 < 15%
Luminal B (HER2^−^)	ER^+^ and/or PR^+^, HER2^−^, Ki67 ≥ 15%
Luminal B (HER2^+^)	ER^+^ and/or PR^+^, HER2^+^
HER2 type	ER^−^, PR^−^, HER2^+^
5 negative phenotype	ER^−^, PR^−^, HER2^−^, CK5^−^ and EGFR^−^
Basal phenotype	ER^−^, PR^−^, HER2^−^, CK5^+^ and/or EGFR^+^

*ER* oestrogen receptor, *PR* progesterone receptor, *HER2* Human epidermal growth factor receptor 2, *CK5* cytokeratin 5, and *EGFR* epidermal growth factor receptor

In the present study, FISH was carried out in accordance with the manufacturer’s guidelines, using Dako Histology FISH Accessory Kit K 579911. The TMA-slides (4 µm) were de-waxed and rehydrated, boiled in a microwave oven for 10 min in Pre-Treatment Solution, and cooled for 15 min. Slides were then washed in Wash Buffer (2 × 3 min), and protein digested with Pepsin Solution for 30 min at 37 °C. The slides were washed in Wash buffer (2 × 3 min) and dehydrated in ethanol for 2 min at each concentration (70, 80 and 95%). The slides were then air-dried for 15 min at room temperature. *CCND1* (3 μL, Empire Genomics) and chromosome enumeration probe 11 (CEP11) (1 μL, Abbott/VYSIS) FISH probes were mixed with hybridization buffer (9 μL, Empire Genomics) and placed on the TMA slides. The slides were coverslipped and sealed with coverslip sealant (Dako) before denaturation at 83 °C for 3 min. Hybridization was done overnight at 37 °C in a DAKO Hybridizer. After hybridization, the slides were rinsed in 0.4xSSC/0.3%NP-40 for 2 min at 72 °C, in 2xSSC/0.1%NP-40 for 15 s at room temperature, and then air-dried for 15 min at 37 °C. DAPI (15 μL, VYSIS. Abbott no 06J50-001) was applied to the slides before coverslipping.

### Scoring and Reporting

*CCND1* and CEP11 CNs were counted in a fluorescence microscope (Nikon Eclipse 90i). All available tissue cylinders were examined, and *CCND1* and CEP11 CN in 20 non-overlapping, well-preserved tumour cell nuclei were recorded. We calculated mean *CCND1* and mean CEP11 CN per tumour cell nucleus for each case. To distinguish between low-level CN gain and potential gene amplification, we separated the cases into three subgroups based on mean *CCND1* CN in the primary tumours: mean *CCND1* CN < 4; mean ≥ 4 < 6; and mean ≥ 6. In the analyses and discussion, mean CN ≥ 6 was regarded as high CN. The same categories were applied to CEP11 CN (< 4; ≥ 4 < 6; and ≥ 6). These cut-offs are based on HER2 guidelines [22], and have been used in previous studies of other genes by our group [17, 18, 23, 24]. The study was conducted according to the REMARK criteria for tumour marker reporting [25].

### Statistical Analyses

We used Pearson’s χ^2^ test to compare proportions of patient and tumour characteristics across the different categories of *CCND1* CN and *CCND1*/CEP11 ratio in the primary tumours and lymph node metastases. We estimated cumulative incidence of BC death five and ten years after the primary diagnosis, considering death from other causes a competing event. We used Gray’s test to test for equality between cumulative incidence curves. We estimated hazard ratios (HR) of death from BC with 95% confidence intervals (CI) according to *CCND1* CN status for all BC cases, censoring at time of death from other causes. A separate Cox regression analysis was done for Luminal A and Luminal B (HER2-) cases combined. We also estimated HRs of death by any cause (overall survival) with 95% CI for all BC cases. In the Cox regression analyses, mean *CCND1* CN < 4 was used as reference. Adjustments were made for age, stage, histopathological grade and Ki67 status. No clear violations of proportionality were observed in log-minus-log plots. All statistical tests were two-sided and statistical significance was assessed at the 5% level. We used Stata version 17 (Stata Corp., College Station, TX, USA) in the statistical analyses.

## Results

The mean age at diagnosis was 75.2 years (range 41–96), and mean follow-up after diagnosis was 9.1 years (Table 2). In the study population, 35.4% had died of BC by the end of follow-up, and 54.2% had died from other causes. Thus, most cases were followed until death. The distribution of stage of disease was as follows: 250 patients (47.5%) were stage I, 222 (42.2%) stage II, 29 (5.5%) stage III, and 23 (4.4%) were stage IV. Information regarding stage was missing for two patients.

**Table 2:** Patient and tumour characteristics

	**Total study** **population**	**Mean *CCND1* copy number**				***CCND1*/CEP11 ratio**		
		** < 4**	** ≥ 4 to < 6**	** ≥ 6**	**p value** **(χ**^**2**^**)**	** < 2**	** ≥ 2**	**p value (χ**^**2**^**)**
N (%)	526	439 (83.5)	45 (8.6)	42 (8.0)		456 (86.7)	70 (13.3)	
Mean age at diagnosis, years (SD)	75.2 (8.3)	75.5 (8.3)	73.7 (6.8)	74.4 (9.5)		75.6 (8.2)	73.8 (8.9)	
Mean follow-up, years (SD)	9.1 (7.1)	9.1 (7.2)	8.7 (5.8)	9.7 (7.6)		9.1 (7.2)	9.4 (6.7)	
Deaths from BC (%)	186 (35.4)	154 (35.1)	19 (42.2)	13 (31.0)		161 (35.3)	25 (35.7)	
Deaths from other causes (%)	285 (54.2)	240 (54.7)	23 (51.1)	22 (52.4)		248 (54.4)	37 (52.9)	
**Histologic grade (%)**								
I	55 (10.5)	52 (11.9)	1 (2.2)	2 (4.8)	0.007	52 (11.4)	3 (4.3)	0.13
II	303 (57.6)	260 (59.2)	24 (53.3)	19 (45.2)		263 (57.7)	40 (57.1)	
III	168 (31.9)	127 (28.9)	20 (44.4)	21 (50.0)		141 (30.9)	27 (38.6)	
**Lymph node metastasis (%)**								
Yes	177 (33.7)	143 (32.6)	20 (44.4)	14 (33.3)	0.37	148 (32.5)	29 (41.4)	0.16
No	233 (44.3)	196 (44.7)	17 (37.8)	20 (47.6)		206 (45.2)	27 (38.6)	
Unknown histology	116 (22.1)	100 (22.8)	8 (17.8)	8 (19.1)		102 (22.4)	14 (20.0)	
**Tumor size (%)**								
≤ 2 cm	251 (47.7)	207 (47.2)	22 (48.9)	22 (52.4)	0.72	213 (46.7)	38 (54.3)	0.14
> 2 cm, ≤ 5 cm	94 (17.9)	76 (17.3)	9 (20.0)	9 (21.4)		79 (17.3)	15 (21.4)	
> 5 cm	10 (1.9)	10 (2.3)	0	0		10 (2.2)	0	
Uncertain, but > 2 cm	64 (12.2)	56 (12.8)	3 (6.7)	5 (11.9)		60 (13.2)	4 (5.7)	
Uncertain	107 (20.3)	90 (20.5)	11 (24.4)	6 (14.3)		94 (20.6)	13 (18.6)	
**Stage (%)**								
I	250 (47.5)	209 (47.6)	18 (40.0)	23 (54.8)	0.74	216 (47.4)	34 (48.6)	0.41
II	222 (42.2)	183 (41.7)	23 (51.1)	16 (38.1)		190 (41.7)	32 (45.7)	
III	29 (5.5)	26 (5.9)	1 (2.2)	2 (4.8)		27 (5.9)	2 (2.9)	
IV	23 (4.4)	20 (4.6)	2 (4.4)	1 (2.4)		22 (4.8)	1 (1.4)	
Uncertain	2 (0.4)	1 (0.2)	1 (2.2)	0		1 (0.22)	1 (1.4)	
**ER (%)**								
ER-	75 (14.3)	62 (14.1)	7 (15.6)	6 (14.3)	0.97	70 (15.4)	5 (7.1)	0.066
ER +	449 (85.4)	375 (85.4)	38 (84.4)	36 (85.7)		384 (84.2)	65 (92.9)	
Unknown	2 (0.4)	2 (0.5)	0	0		2 (0.4)	0	
**Molecular subtype (%)**								
Luminal A	284 (54.0)	253 (57.6)	16 (35.6)	15 (35.7)	0.002	253 (55.5)	31 (44.3)	0.02
Luminal B (HER2-)	126 (24.0)	89 (20.3)	17 (37.8)	20 (47.6)		99 (21.7)	27 (38.6)	
Luminal B (HER2 +)	41 (7.8)	35 (8.0)	5 (11.1)	1 (2.4)		34 (7.5)	7 (10.0)	
HER2 type	25 (4.8)	19 (4.3)	3 (6.7)	3 (7.1)		22 (4.8)	3 (4.3)	
5 negative phenotype	12 (2.3)	11 (2.5)	0	1 (2.4)		11 (2.4)	1 (1.4)	
Basal phenotype	38 (7.2)	32 (7.3)	4 (8.9)	2 (4.8)		37 (8.1)	1 (1.4)	
**Histological subtype (%)**								
Ductal carcinoma	363 (69.0)	304 (69.3)	34 (75.6)	25 (59.5)	0.056	317 (69.5)	46 (65.7)	0.14
Lobular carcinoma	71 (13.5)	58 (13.2)	5 (11.1)	8 (19.1)		60 (13.2)	11 (15.7)	
Tubular carcinoma	1 (0.2)	1 (0.2)	0	0		1 (0.2)	0	
Mucinous carcinoma	25 (4.8)	24 (5.5)	0	1 (2.4)		23 (5.0)	2 (2.9)	
Medullary carcinoma	13 (2.5)	7 (1.6)	1 (2.2)	5 (11.9)		8 (1.8)	5 (7.1)	
Papillary carcinoma	24 (4.6)	21 (4.8)	2 (4.4)	1 (2.4)		22 (4.8)	2 (2.9)	
Metaplastic carcinoma	9 (1.7)	8 (1.8)	1 (2.2)	0		9 (2.0)	0	
Other	20 (3.8)	16 (3.6)	2 (4.4)	2 (4.8)		16 (3.5)	4 (5.7)	
**Ki67 high/low (%)**								
Ki67 < 15%	320 (60.8)	286 (65.2)	17 (37.8)	17 (40.5)	< 0.0001	287 (62.9)	33 (47.1)	0.012
Ki67 ≥ 15%	206 (39.2)	153 (34.9)	28 (62.2)	25 (59.5)		169 (37.1)	37 (52.9)	
**Mitoses/10 HPF, median (IQR p25, p75)**	5 (1, 12)	4 (1, 10)	9 (2, 17)	8.5 (3, 20)		4.5 (1, 11)	8 (2, 17)	
**Mitoses/10 HPF, quartiles (%)**								
≤ 1	143 (27.2)	127 (28.9)	10 (22.2)	6 (14.3)	0.006	130 (28.5)	13 (18.6)	0.03
> 1, ≤ 5	137 (26.1)	121 (27.6)	8 (17.8)	8 (19.1)		123 (27.0)	14 (20.0)	
> 5, ≤ 12	125 (23.8)	104 (23.7)	11 (24.4)	10 (23.8)		107 (23.5)	18 (25.7)	
> 12	121 (23.0)	87 (19.8)	16 (35.6)	18 (42.9)		96 (21.1)	25 (35.7)	

*SD* standard deviation, *BC* breast cancer, *HER2* human epidermal growth factor receptor 2

### *CCND1 *and* CEP11* Copy Numbers in Primary Tumours

**Fig. 2:**
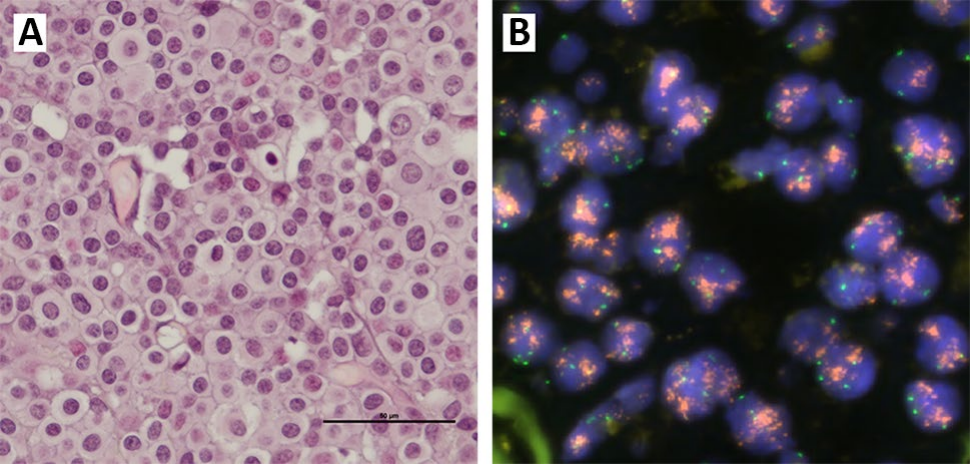
(A) HES-stained tissue section from tumour tissue (× 400 magnification). (B) Fluorescence in situ hybridization for *CCND1* and CEP11 showing increased copy number of *CCND1* (red) and 2–4 copies of CEP11 (green)

*CCND1 c*opy CN increase (mean ≥ 4) was observed in 87 cases (16.6%). We found mean *CCND1* CN ≥ 4 < 6 in 45 cases (8.6%), and mean CN ≥ 6 in 42 cases (8.0%)., In tumours with CN increase, CN increase was observed in most tumour cells (Fig. 2). Nine cases (2%) had mean CEP11 CN ≥ 4 < 6, and three (0.6%) had mean CEP11 CN ≥ 6. Of the 42 cases with mean *CCND1* CN ≥ 6, none had mean CEP11 CN ≥ 6. Thus, high *CCND1* CN was not accompanied by an increase in CEP11 CN (Table 3). Seventy cases (13.3%) had *CCND1*/CEP11 ratio ≥ 2.

**Table 3:** *CCND1* and CEP11 copy number in primary tumors

	**Mean *CCND1* copy number (%)**				
	** < 4**	** ≥ 4 < 6**	** ≥ 6**	**Total**	**p value (χ**^**2**^**)**
**Mean CEP11 copy number (%)**					
**< 4**	436 (99.3)	40 (88.9)	38 (90.5)	514	p < 0.0001
**≥ 4 < 6**	2 (0.5)	3 (6.7)	4 (9.5)	9	
**≥ 6**	1 (0.2)	2 (4.4)	0	3	
Total	439	45	42	526	

### *CCND1* and Molecular and Histopathological Subtypes, Histopathological Grade, and Proliferation

High mean *CCND1* CN (mean ≥ 6) was seen among all molecular subtypes. The highest proportion of cases with high mean *CCND1* CN was seen among Luminal B (HER2^−^) cases (20/126; 16%). High *CCND1* CN was seen among all histopathological subtypes except tubular and metaplastic carcinomas. However, the study population only included one case of tubular carcinoma and nine cases of metaplastic carcinomas (Table 2). The highest proportion of cases with high *CCND1* CN was seen among medullary carcinomas (5/13; 38%). The proportion of cases with histopathological grade 3 tumours was higher among cases with mean *CCND1* CN ≥ 6, compared to cases with mean CN < 4 (50% vs. 29%, p = 0.007).

There was an association between increased *CCND1* CN and proliferation. Of the cases with mean CN ≥ 4 < 6, 28/45 (62%) were Ki67 high (≥ 15% Ki67 positive cells), and with mean CN ≥ 6, 25/42 (59.5%) were Ki67 high, compared to 153/439 (35%) among cases with mean CN < 4 (p < 0.0001). Mitotic counts were higher among cases with mean *CCND1* CN ≥ 6, compared to cases with mean CN < 4 (43% vs. 20% in the upper quartile, respectively, p = 0.006).

*CCND1*/CEP11 ratio ≥ 2 was seen among all molecular subtypes. The highest proportion of cases with ratio ≥ 2 was seen among Luminal B (HER2^−^) cases (27/126; 21%). Ratio ≥ 2 was seen among all histopathological subtypes except tubular and metaplastic carcinomas. The highest proportion of cases with ratio ≥ 2 was seen among medullary carcinomas (5/13; 38%). There was no clear association between *CCND1*/CEP11 ratio and histopathological grade.

There was an association between *CCND1*/CEP11 ratio and proliferation. Of cases with ratio ≥ 2, 37 (53%) were Ki67 high, whereas 169 (37%) cases with ratio < 2 were Ki67 high (p = 0.012). Mitotic counts were also higher among cases with ratio ≥ 2, compared to cases with ratio < 2 (36% vs. 21% in the upper quartile, p = 0.03).

### *CCND1*, *FGFR1* and *ZNF703*

Among the 526 patients included in this study, *FGFR1* and *ZNF703* CN status was also available for 507 cases [17, 18]. For 495 patients, copy number of all three markers was available. Of the 507 cases with *CCND1* and *FGFR1* copy number status available, 40 patients had high *CCND1* CN (mean ≥ 6). Six of the 40 cases (15.0%) also had mean *FGFR1* CN ≥ 6, and of these, two were histopathological grade 2 and four were grade 3. Furthermore, five of the six cases with high *CCND1* and *FGFR1* CNs were Ki67 high and four had mitotic counts in the upper quartile. One case was Luminal A, and five were Luminal B (HER2^−^). Of the 507 cases with *CCND1* and *ZNF703* copy number status available, 38 had high *CCND1* CN. Five of the 38 cases (13.2%) also had mean *ZNF703* CN ≥ 6 (Table 4) and of these, one tumour was histopathological grade 2 and four were grade 3. Furthermore, three of the five were Ki67 high and three had mitotic counts in the upper quartile. One case was Luminal A, three were Luminal B (HER2^−^), and one was HER2 type. Three patients had tumours with high CNs of all three genes. These three tumours were histopathological grade 3, Luminal B (HER2^−^), had high Ki67 levels and mitotic counts in the upper quartile.

**Table 4:** *CCND1, FGFR1* and *ZNF703* copy numbers in primary tumours

	**Mean *CCND1*/tumour cell (%)**				
	** < 4**	** ≥ 4, < 6**	** ≥ 6**	**Total**	**p value (χ**^**2**^**)**
**Mean *FGFR1*/tumor cell (%)**					
< 4	378 (89.2)	29 (67.4)	30 (75.0)	437	< 0.0001
≥ 4, < 6	22 (5.2)	4 (9.3)	4 (10.0)	30	
≥ 6	24 (5.7)	10 (23.3)	6 (15.0)	40	
Total	424	43	40	507	
**Mean *ZNF703*/tumor cell (%)**					
< 4	385 (90.6)	26 (59.1)	30 (79.0)	441	< 0.0001
≥ 4, < 6	20 (4.7)	9 (20.5)	3 (7.9)	32	
≥ 6	20 (4.7)	9 (20.5)	5 (13.2)	34	
Total	425	44	38	507	

### *CCND1* in Lymph Node Metastases

High mean *CCND1* CN (mean ≥ 6) in the primary tumour was most often also followed by a concurrent CN increase in the corresponding lymph node metastasis. Of the 123 cases with available lymph node metastases, 11 (9%) had high mean *CCND1* CN in their primary tumours (Table 5), and all but one also had high mean CN in the corresponding lymph node metastasis.

**Table 5:** *CCND1* mean copy number in primary tumors and corresponding lymph node metastases

	**Mean *CCND1*, primary tumors**			
	< 4	≥ 4, < 6	≥ 6	Total
**Mean *CCND1*, lymph nodes**				
< 4	94 (97.9)	6 (37.5)	1 (9.1)	101
≥ 4, < 6	2 (2.1)	6 (37.5)	0	8
≥ 6	0	4 (25.0)	10 (90.9)	14
Total	96	16	11	123

### *CCND1* and Prognosis

**Fig. 3:**
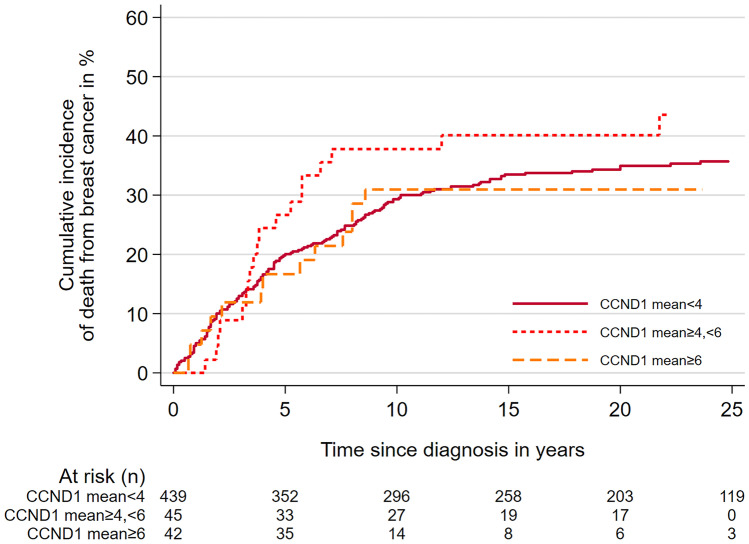
Cumulative incidence of death from breast cancer according to mean copy number of *CCND1* (Gray’s test p = 0.5)

There was no association between high *CCND1* CN, and a poor prognosis. After 10 years of follow-up, the cumulative incidence of death from BC was 31% (95% CI 19–47) among cases with mean CN ≥ 6, 38% (95% CI 26–54) for mean CN ≥ 4 < 6, and 29% (95% CI 25–34) for mean CN < 4 (Fig. 3). In the Cox regression analyses using mean *CCND1* CN < 4 as the reference, we found that the rate of death from BC was similar for cases with mean *CCND1* CN ≥ 6 (HR 0.82 (95% CI 0.5–1.4), Table 6).

**Table 6:** Absolute and relative risk of death from breast cancer and overall survival according to mean *CCND1* copy number in primary tumours

	**Mean *CCND1 copy number***		
	** < 4**	** ≥ 4 < 6**	** ≥ 6**
**Breast cancer specific death, all cases**			
Cum. risk after 5 years (%) (95% CI)	19.8 (16.4–23.9)	26.7 (16.1–42.2)	16.7 (8.3–31.8)
Cum. risk after 10 years (%) (95% CI)	29.3 (25.3–33.8)	37.8 (25.5–53.5)	31.0 (19.3–47.3)
HR, unadjusted (95% CI)	1.0	1.21 (0.76–1.97)	0.82 (0.46–1.44)
HR adjusted for age (95% CI)	1.0	1.21 (0.75–1.96)	0.85 (0.48–1.51)
HR adjusted for stage (95% CI)	1.0	1.14 (0.70–1.86)	0.99 (0.56–1.75)
HR adjusted for grade (95% CI)	1,0	1.11 (0.68–1.79)	0.75 (0.42–1.32)
HR adjusted for Ki67 (95% CI)	1.0	1.01 (0.62–1.65)	0.67 (0.37–1.18)
HR adjusted for age, stage, grade, Ki67 (95% CI)	1.0	0.97 (0.59–1.61)	0.79 (0.44–1.44)
**Overall survival, all cases**			
HR, unadjusted (95% CI)	1.0	1.12 (0.81–1.54)	0.86 (0.61–1.22)
HR adjusted for age (95% CI)	1.0	1.06 (0.77–1.46)	0.91 (0.64–1.30)
HR adjusted for stage (95% CI)	1.0	1.13 (0.82–1.57)	0.93 (0.65–1.31)
HR adjusted for grade (95% CI)	1.0	1.06 (0.77–1.47)	0.83 (0.59–1.17)
HR adjusted for Ki67 (95% CI)	1.0	1.07 (0.77–1.48)	0.81 (0.57–1.15)
HR adjusted for age, stage, grade, Ki67 (95% CI)	1.0		
**Breast cancer specific death,** **Luminal A and Luminal B (HER2**^**−**^**) cases**			
HR, unadjusted (95% CI)	1.0	1.26 (0.70–2.30)	0.95 (0.51–1.78)
HR adjusted for age (95% CI)	1.0	1.27 (0.70–2.31)	1.04 (0.55–1.96)
HR adjusted for stage (95% CI)	1.0	1.31 (0.72–2.39)	1.09 (0.58–2.05)
HR adjusted for grade (95% CI)	1.0	1.14 (0.62–2.09)	0.85 (0.45–1.61)
HR adjusted for age, stage, grade (95% CI)	1.0	1.25 (0.68–2.29)	1.01 (0.52–1.97)

*CI* confidence interval, *HR* hazard ratio, *HER2* Human epidermal growth factor receptor 2

Similar results were seen in analyses of death from any causes. In the Cox regression analyses using mean *CCND1* CN < 4 as the reference, the rate of death was similar for cases with mean *CCND1* CN ≥ 6 (HR 0.86 (95% CI 0.6–1.2)) (Table 6).

In the Cox regression analysis restricted to Luminal A and Luminal B (HER2^−^) cases (n = 410) using mean *CCND1* CN < 4 as the reference, the rate of death from BC was similar for cases with mean *CCND1* CN ≥ 6 (HR 0.95 (95% CI 0.51–1.78), Table 6).

## Discussion

In this study, we found that 42 (8.0%) of the cases had high mean *CCND1* CN (mean ≥ 6). The largest proportion of cases with high *CCND1* CN was found among medullary carcinomas and Luminal B (HER2^−^) tumours. There was an association between high *CCND1* CN, and high histopathological grade and proliferation, but no association with a poor prognosis. There was good concordance between *CCND1* CN increase in the primary tumours and in the corresponding lymph node metastases. Of the primary BCs with high *CCND1* CN for which *FGFR1* CN status was available, 15.0% also had high *FGFR1* CN. Similarly, 13.2% has high *ZNF703* CN. Three cases showed high CN of all three genes.

The cases in this study came from a large, well-described cohort of Norwegian BC patients with unusually long-term follow-up. Since BC recurrence and death can occur decades after the primary diagnosis, long follow-up is of great value in studies of prognostic markers. The cases in this study were identified and followed through linkage with high quality, national registries [26, 27]. Histopathological typing and grading were done by two experienced pathologists, and molecular subtyping was done using the same algorithm, antibodies and cut-off levels for all cases [20]. Contrary to multigene assays, an in situ method such as FISH is readily available in most laboratories, and when applied to tissue microarrays, offers us the opportunity to study individual biomarkers in large numbers of samples at a relatively low cost. More importantly, FISH ensures that only invasive tumour cells are included for assessment. However, using FISH on tissue sections, may result in signal truncation loss and consequently, an underestimation of CNs compared to analysis of whole nuclei [28, 29]. The cases were diagnosed over a time span of several decades, and preanalytical conditions will have varied, possibly affecting the number of cases suited for FISH analysis, however few cases were excluded for our series due to unsuccessful FISH. Similarly, treatment protocols varied over time and information on individual treatment was unavailable to us. Patients diagnosed early in the study period would have received surgery only which would have been the standard treatment at the time of diagnosis. Others diagnosed later may not have qualified for further treatment due to their age at diagnosis. This allows us to study the effect of our findings on long-term outcome in a population of patients with few other treatment interventions beyond surgery. In the analyses, some subgroups were small, and therefore the results should be interpreted accordingly.

We found high *CCND1* CN (mean ≥ 6) in 8% of BCs. This is similar to findings in a number of other *CCND1* CN studies [9, 12, 13]. However, a study using next generation sequencing found *CCND1* amplification in 15% of patients [11]. There are no established guidelines for the assessment of *CCND1* CN, and our choice of cut-off values was based on HER2 guidelines [22] and previous studies by our group [17, 18, 23, 24]. For HER2, the use of HER2/CEP17 ratio for clinical decision-making has been the subject of some debate. However, it has been shown that CEP17 enumeration may be of value in a small number of cases exhibiting chromosome 17 aneusomy in which gene CN alone may falsely under- or overestimate amplification status [29]. Due to the uncertainty truncation artefacts confers on studies of gene loss, we did not estimate gene deletion in this study. We found that *CCND1* CN increase was rarely accompanied by increase in CEP11 CN, and thus the use of ratio in addition to *CCND1* mean CN in the analyses may not provide additional information. We did not include *CCND1*/CEP11 ratio in our analyses of prognosis.

Similar to other studies, we found an association between *CCND1* amplification, and high histopathological grade, high proliferation [9, 12] and the Luminal B subtype [9]. This is in agreement with Curtis et al*.*, who described a high-risk oestrogen receptor positive 11q13/14 subgroup of BC comprising a number of genes exhibiting high CN aberrations, including *CCND1* [30] In the present study, while high *CCND1* CN was most frequent in the Luminal B (HER2^−^)subtype (20/126 cases), it was also observed among Luminal A tumours (15/284 cases). Only seven cases showed high CN among the remaining molecular subtypes.

Several studies have shown that high *CCND1* CN is associated with risk of recurrence [9, 12, 31],while others demonstrate an association with disease specific survival [32, 33], but not relapse free survival. We found no association between *CCND1* amplification and risk of death from BC. While the proportions of cases with high *CCND1* CN are similar across studies, including our own, differences in patient populations may explain the varying results. Our patient series comprised women with a relatively high mean age compared to other studies [9, 32]. Methodological issues such as varying cut-off levels in the interpretation of CN may also account for differing results.

Ortiz et al*.* found that the influence of *CCND1* amplification on risk of recurrence was restricted to tumours with high amplification (defined as > 10 copies of the gene). In our study population, 13 of the 42 patients with high CN had > 10 copies. Cox regression analysis comparing this subgroup to patients without CN increase (mean < 4) identified no association with prognosis. *CCND1* amplification has been shown to be associated with Cyclin D1 protein expression, and the prognostic influence of the protein may be subtype specific [9]. To clarify the potential prognostic impact of *CCND1* CN increase it would be of value to study a larger BC cohort with clinical data on disease free survival in addition to disease specific survival, enabling subtype specific prognostic analyses of the role of *CCND1* in BC progression and prognosis. Furthermore, a correlation of data from in situ analysis of *CCND1* CN and cyclin D1 protein expression by IHC in the prognostic analyses could be of interest.

Some BCs exhibiting *CCND1* amplification show concomitant amplification of genes located on chromosome 8, such as *FGFR1* [14] and *ZNF703* [15]. *FGFR1* and ZNF703 are both located on 8p11.23 [3], but amplification of one of the two genes is not necessarily accompanied by amplification of the other [34]. *FGFR1* encodes Fibroblast growth factor receptor 1 which is involved in the regulation of cell proliferation, differentiation, and survival [35]. *ZNF703* encodes Zinc finger protein 703 which regulates cell adhesion, migration and proliferation, and the cellular response to estradiol stimulus [36].

Amplification of *FGFR1* has been found in 8% to 15% of BCs [17, 37, 38] and, in similarity to *CCND1*, it is associated with the Luminal B subtype of BC [17, 39]. *FGFR1* amplification is associated with proliferation and a poor prognosis [16, 40], especially in ER positive BC [17, 37, 41]. *ZNF703* amplification and overexpression are associated with high proliferation [18, 36, 42, 43] and the Luminal B subtype [36, 42, 44, 45]. In luminal tumours, *ZNF703* amplification and overexpression is associated with a poor prognosis [36, 42].

In our study, six of 40 patients (15.0%) with high *CCND1* CN also had mean *FGFR1* CN ≥ 6. Of these, five were Luminal B (HER2^−^) and one was Luminal A, five were Ki67 high and four had high mitotic counts. Five of the 38 cases (13.2%) with high *CCND1* CN also had high *ZNF703* CN. Of these, one was histopathological grade 2 and four were histopathological grade 3. One was Luminal A, three were Luminal B (HER2^−^), and one was HER2 type. Furthermore, three were Ki67 high and three had high mitotic counts. Our findings confirm that these three genes are associated with highly proliferative, oestrogen receptor-positive BC.

There were 123 cases with lymph node metastases. There was complete agreement with regard to *CCND1* copy number in 110 cases. Thirteen cases had discrepant results. Only one case showed high CN in the primary tumour and normal CN in the lymph node metastasis. There may be a biological explanation for this but equally, the explanation may lie in the method. Tissue microarrays are small samples of larger tumour masses and tumour heterogeneity could explain the discrepant results [46]. In this study the number of cases with lymph node metastases and *CCND1* status may be too low to enable us to draw reliable conclusions.

Amplification of *CCND1* and *FGFR1* and/or *ZNF703* can occur due to translocation, or to other genetic changes [34]. *CCND1* CN increase can therefore result from different molecular events, potentially involving other genes, thus complicating the assessment of the prognostic impact of *CCND1* CN increase alone [34]. Amplification of chromosomal regions 8p12 and 11q13 are frequent in BC and are often associated with oestrogen receptor positive tumours [47]. It has been shown that tumours coamplified for *FGFR1* and *CCND1* are associated with an especially poor prognosis [48]. Kwek et al*.* suggested that genes located on the 8p12 amplicon including *FGFR1* and *ZNF703*, and CCND1 on 11q13, cooperate with each other in major oncogenic pathways but that the numbers of genes involved in these pathways and the complexity of their cross-talk remains to be clarified [49]. Therefore, a study of the potential prognostic influence of coamplification would be of great value. Thus, multigene assays in large cohorts may be necessary to clarify the potential role of *CCND1* as a prognostic marker.

Breast tumorigenesis is strongly dependent on the oestrogen-ER signaling pathway and consequently endocrine treatment has been the treatment of choice for ER-positive BC for decades. However, approximately 30% of patients develop endocrine resistance [50]. Both *CCND1* and *FGFR1* have been shown to be associated with endocrine resistance [51–55]. Thus, a deeper understanding of the roles these genes play in BC with regard to endocrine resistance should be of clinical relevance.

## Conclusion

High *CCND1* CN occurs across all molecular subtypes, but most frequently in the Luminal B (HER2^−^) subtype. It is associated with aggressive tumour features such as high histopathological grade, and high proliferation. There was good correlation between primary tumours and axillary lymph node metastases with regard to *CCND1* CN. The prognostic value of high *CCND1* copy number in BC tumours remains unresolved.

## Data Availability

The datasets generated during and/or analysed during the current study are not publicly available due to reasons of sensitivity and limitations imposed in the conditions for approval by the Ethics Committee but are available from the corresponding author on reasonable request.

## References

[1] World Health Organization (WHO) International Agency for Research on Cancer: Cancer today 2021 [cited 06 May 2021]. Available from: https://gco.iarc.fr/today.

[2] Wang LC, Chen HM, Chen JH, Lin YC, Ko Y. An evaluation of the healthcare costs associated with adverse events in patients with breast cancer. Int J Health Plann Manage. 2021 Sep;36(5):1465–75. 10.1002/hpm.3184. 10.1002/hpm.3184 33914358

[3] Genecards. The Human Gene Database. 2016. Available from: www.genecards.org.

[4] Sherr CJ (1994). G1 phase progression: cycling on cue. Cell.

[5] Hinds PW, Dowdy SF, Eaton EN, Arnold A, Weinberg RA (1994). Function of a human cyclin gene as an oncogene. Proc Natl Acad Sci U S A.

[6] Neuman E, Ladha MH, Lin N, Upton TM, Miller SJ, DiRenzo J (1997). Cyclin D1 stimulation of estrogen receptor transcriptional activity independent of cdk4. Mol Cell Biol.

[7] Zwijsen RM, Wientjens E, Klompmaker R, van der Sman J, Bernards R, Michalides RJ (1997). CDK-independent activation of estrogen receptor by cyclin D1. Cell.

[8] Petre-Draviam CE, Cook SL, Burd CJ, Marshall TW, Wetherill YB, Knudsen KE (2003). Specificity of cyclin D1 for androgen receptor regulation. Cancer Res.

[9] Ortiz AB, Garcia D, Vicente Y, Palka M, Bellas C, Martin P (2017). Prognostic significance of cyclin D1 protein expression and gene amplification in invasive breast carcinoma. PLoS ONE.

[10] Bartkova J, Lukas J, Müller H, Lützhøft D, Strauss M, Bartek J (1994). Cyclin D1 protein expression and function in human breast cancer. Int J Cancer.

[11] Yang L, Ye F, Bao L, Zhou X, Wang Z, Hu P (2019). Somatic alterations of TP53, ERBB2, PIK3CA and CCND1 are associated with chemosensitivity for breast cancers. Cancer Sci.

[12] Lundgren K, Brown M, Pineda S, Cuzick J, Salter J, Zabaglo L (2012). Effects of cyclin D1 gene amplification and protein expression on time to recurrence in postmenopausal breast cancer patients treated with anastrozole or tamoxifen: a TransATAC study. Breast Cancer Res.

[13] Bieche I, Olivi M, Nogues C, Vidaud M, Lidereau R (2002). Prognostic value of CCND1 gene status in sporadic breast tumours, as determined by real-time quantitative PCR assays. Br J Cancer.

[14] Bautista S, Theillet C (1998). CCND1 and FGFR1 coamplification results in the colocalization of 11q13 and 8p12 sequences in breast tumor nuclei. Genes Chromosomes Cancer.

[15] Ooi A, Inokuchi M, Horike SI, Kawashima H, Ishikawa S, Ikeda H (2019). Amplicons in breast cancers analyzed by multiplex ligation-dependent probe amplification and fluorescence in situ hybridization. Hum Pathol.

[16] Fumagalli D, Wilson TR, Salgado R, Lu X, Yu J, O'Brien C (2016). Somatic mutation, copy number and transcriptomic profiles of primary and matched metastatic estrogen receptor-positive breast cancers. Ann Oncol.

[17] Bofin AM, Ytterhus B, Klæstad E, Valla M. FGFR1 copy number in breast cancer: associations with proliferation, histopathological grade and molecular subtypes. J Clin Pathol. 2021 Mar 22. 10.1136/jclinpath-2021-207456. 10.1136/jclinpath-2021-207456 33753561

[18] Klæstad E, Sawicka JE, Engstrøm MJ, Ytterhus B, Valla M, Bofin AM (2021). ZNF703 gene copy number and protein expression in breast cancer; associations with proliferation, prognosis and luminal subtypes. Breast Cancer Res Treat.

[19] Kvale G, Heuch I, Eide GE (1987). A prospective study of reproductive factors and breast cancer. I Parity Am J Epidemiol.

[20] Engstrom MJ, Opdahl S, Hagen AI, Romundstad PR, Akslen LA, Haugen OA (2013). Molecular subtypes, histopathological grade and survival in a historic cohort of breast cancer patients. Breast Cancer Res Treat.

[21] Lakhani SR, Ellis IO, Schnitt SJ, Tan PH, van de Vijver MJ, editors. WHO Classification of Tumours of the Breast. 4th ed. Lyon: International Agency for Research on Cancer (IARC). 2012.

[22] Wolff AC, Hammond MEH, Allison KH, Harvey BE, Mangu PB, Bartlett JMS (2018). Human Epidermal Growth Factor Receptor 2 Testing in Breast Cancer: American Society of Clinical Oncology/College of American Pathologists Clinical Practice Guideline Focused Update. J Clin Oncol.

[23] Valla M, Opdahl S, Ytterhus B, Bofin AM (2021). DTX3 copy number increase in breast cancer: a study of associations to molecular subtype, proliferation and prognosis. Breast Cancer Res Treat.

[24] Klæstad E, Opdahl S, Engstrøm MJ, Ytterhus B, Wik E, Bofin AM (2020). MRPS23 amplification and gene expression in breast cancer; association with proliferation and the non-basal subtypes. Breast Cancer Res Treat.

[25] McShane LM, Altman DG, Sauerbrei W, Taube SE, Gion M, Clark GM (2006). REporting recommendations for tumor MARKer prognostic studies (REMARK). Breast Cancer Res Treat.

[26] Larsen IK, Smastuen M, Johannesen TB, Langmark F, Parkin DM, Bray F (2009). Data quality at the Cancer Registry of Norway: an overview of comparability, completeness, validity and timeliness. Eur J Cancer.

[27] Norwegian Cause of Death Registry [Available from: www.fhi.no/en/hn/health-registries/cause-of-death-registry/.

[28] Yoshimoto M, Ludkovski O, Good J, Pereira C, Gooding RJ, McGowan-Jordan J (2018). Use of multicolor fluorescence in situ hybridization to detect deletions in clinical tissue sections. Lab Invest.

[29] Bartlett JM, Campbell FM, Mallon EA (2008). Determination of HER2 amplification by in situ hybridization: when should chromosome 17 also be determined?. Am J Clin Pathol.

[30] Curtis C, Shah SP, Chin SF, Turashvili G, Rueda OM, Dunning MJ (2012). The genomic and transcriptomic architecture of 2,000 breast tumours reveals novel subgroups. Nature.

[31] Bostner J, Ahnström Waltersson M, Fornander T, Skoog L, Nordenskjöld B, Stål O (2007). Amplification of CCND1 and PAK1 as predictors of recurrence and tamoxifen resistance in postmenopausal breast cancer. Oncogene.

[32] Elsheikh S, Green AR, Aleskandarany MA, Grainge M, Paish CE, Lambros MB (2008). CCND1 amplification and cyclin D1 expression in breast cancer and their relation with proteomic subgroups and patient outcome. Breast Cancer Res Treat.

[33] Lundberg A, Lindström LS, Li J, Harrell JC, Darai-Ramqvist E, Sifakis EG (2019). The long-term prognostic and predictive capacity of cyclin D1 gene amplification in 2305 breast tumours. Breast Cancer Res.

[34] Paterson AL, Pole JC, Blood KA, Garcia MJ, Cooke SL, Teschendorff AE (2007). Co-amplification of 8p12 and 11q13 in breast cancers is not the result of a single genomic event. Genes Chromosomes Cancer.

[35] Ornitz DM, Xu J, Colvin JS, McEwen DG, MacArthur CA, Coulier F (1996). Receptor specificity of the fibroblast growth factor family. J Biol Chem.

[36] Sircoulomb F, Nicolas N, Ferrari A, Finetti P, Bekhouche I, Rousselet E (2011). ZNF703 gene amplification at 8p12 specifies luminal B breast cancer. EMBO Mol Med.

[37] Elbauomy Elsheikh S, Green AR, Lambros MB, Turner NC, Grainge MJ, Powe D (2007). FGFR1 amplification in breast carcinomas: a chromogenic in situ hybridisation analysis. Breast Cancer Res.

[38] Theillet C, Adelaide J, Louason G, Bonnet-Dorion F, Jacquemier J, Adnane J (1993). FGFRI and PLAT genes and DNA amplification at 8p12 in breast and ovarian cancers. Genes Chromosomes Cancer.

[39] Mouron S, Manso L, Caleiras E, Rodriguez-Peralto JL, Rueda OM, Caldas C (2021). FGFR1 amplification or overexpression and hormonal resistance in luminal breast cancer: rationale for a triple blockade of ER, CDK4/6, and FGFR1. Breast Cancer Res.

[40] Bourrier C, Pierga J-Y, Xuereb L, Salaun H, Proudhon C, Speicher MR (2020). Shallow Whole-Genome Sequencing from Plasma Identifies FGFR1 Amplified Breast Cancers and Predicts Overall Survival. Cancers (Basel).

[41] Jang MH, Kim EJ, Choi Y, Lee HE, Kim YJ, Kim JH (2012). FGFR1 is amplified during the progression of in situ to invasive breast carcinoma. Breast Cancer Res.

[42] Holland DG, Burleigh A, Git A, Goldgraben MA, Perez-Mancera PA, Chin SF (2011). ZNF703 is a common Luminal B breast cancer oncogene that differentially regulates luminal and basal progenitors in human mammary epithelium. EMBO Mol Med.

[43] Slorach EM, Chou J, Werb Z (2011). Zeppo1 is a novel metastasis promoter that represses E-cadherin expression and regulates p120-catenin isoform expression and localization. Genes Dev.

[44] Reynisdottir I, Arason A, Einarsdottir BO, Gunnarsson H, Staaf J, Vallon-Christersson J (2013). High expression of ZNF703 independent of amplification indicates worse prognosis in patients with luminal B breast cancer. Cancer Med.

[45] Głodzik D, Purdie C, Rye IH, Simpson PT, Staaf J, Span PN (2018). Mutational mechanisms of amplifications revealed by analysis of clustered rearrangements in breast cancers. Ann Oncol.

[46] Pinder SE, Brown JP, Gillett C, Purdie CA, Speirs V, Thompson AM (2013). The manufacture and assessment of tissue microarrays: suggestions and criteria for analysis, with breast cancer as an example. J Clin Pathol.

[47] Letessier A, Sircoulomb F, Ginestier C, Cervera N, Monville F, Gelsi-Boyer V (2006). Frequency, prognostic impact, and subtype association of 8p12, 8q24, 11q13, 12p13, 17q12, and 20q13 amplifications in breast cancers. BMC Cancer.

[48] Cuny M, Kramar A, Courjal F, Johannsdottir V, Iacopetta B, Fontaine H (2000). Relating Genotype and Phenotype in Breast Cancer: An Analysis of the Prognostic Significance of Amplification at Eight Different Genes or Loci and of *p53* Mutations. Cancer Res.

[49] Kwek SS, Roy R, Zhou H, Climent J, Martinez-Climent JA, Fridlyand J (2009). Co-amplified genes at 8p12 and 11q13 in breast tumors cooperate with two major pathways in oncogenesis. Oncogene.

[50] Dimitrakopoulos FI, Kottorou A, Tzezou A (2021). Endocrine resistance and epigenetic reprogramming in estrogen receptor positive breast cancer. Cancer Lett.

[51] Haque MM, Desai KV (2019). Pathways to Endocrine Therapy Resistance in Breast Cancer. Front Endocrinol (Lausanne).

[52] Rani A, Stebbing J, Giamas G, Murphy J (2019). Endocrine Resistance in Hormone Receptor Positive Breast Cancer-From Mechanism to Therapy. Front Endocrinol (Lausanne)..

[53] Turner N, Pearson A, Sharpe R, Lambros M, Geyer F, Lopez-Garcia MA (2010). FGFR1 amplification drives endocrine therapy resistance and is a therapeutic target in breast cancer. Cancer Res.

[54] Prall OW, Rogan EM, Musgrove EA, Watts CK, Sutherland RL (1998). c-Myc or cyclin D1 mimics estrogen effects on cyclin E-Cdk2 activation and cell cycle reentry. Mol Cell Biol.

[55] Hanker AB, Sudhan DR, Arteaga CL (2020). Overcoming Endocrine Resistance in Breast Cancer. Cancer Cell.

